# Targeted escape of SARS-CoV-2 *in vitro* from monoclonal antibody S309, the precursor of sotrovimab

**DOI:** 10.3389/fimmu.2022.966236

**Published:** 2022-08-24

**Authors:** Clara Luzia Magnus, Andreas Hiergeist, Philipp Schuster, Anette Rohrhofer, Jan Medenbach, André Gessner, David Peterhoff, Barbara Schmidt

**Affiliations:** ^1^ Institute of Clinical Microbiology and Hygiene, University Hospital Regensburg, Regensburg, Germany; ^2^ Institute of Medical Microbiology and Hygiene, University of Regensburg, Regensburg, Germany; ^3^ Biochemistry I, Faculty of Biology and Pre-Clinical Medicine, University of Regensburg, Regensburg, Germany

**Keywords:** SARS-CoV-2, receptor-binding domain, monoclonal antibody, sotrovimab, immune escape, entry, endocytosis, fusion

## Abstract

Class 1 and 2 monoclonal antibodies inhibit SARS-CoV-2 entry by blocking the interaction of the viral receptor-binding domain with angiotensin-converting enzyme 2 (ACE2), while class 3 antibodies target a highly conserved epitope outside the ACE2 binding site. We aimed to investigate the plasticity of the spike protein by propagating wild-type SARS-CoV-2 in the presence of class 3 antibody S309. After 12 weeks, we obtained a viral strain that was completely resistant to inhibition by S309, due to successively evolving amino acid exchanges R346S and P337L located in the paratope of S309. The antibody lost affinity to receptor-binding domains carrying P337L or both amino acid exchanges, while ACE2 binding was not affected. The resistant strain replicated efficiently in human CaCo-2 cells and was more susceptible to inhibition of fusion than the original strain. Overall, SARS-CoV-2 escaped inhibition by class 3 antibody S309 through a slow, but targeted evolution enabling immune escape and altering cell entry. This immune-driven enhancement of infectivity and pathogenicity could play an important role in the future evolution of SARS-CoV-2, which is under increasing immunological pressure from vaccination and previous infections.

## Introduction

A growing number of monoclonal antibodies has been approved for the prophylactic and therapeutic use in people at risk for a severe course of coronavirus disease 2019 (COVID-19). Class 1 and class 2 (e.g. Regeneron) antibodies are blocking the interaction with angiotensin-converting enzyme type 2 (ACE2) by binding to the ‘up’ and ‘up and down’ conformation of the viral receptor-binding domain (RBD); class 3 (e.g. S309) is neutralizing by binding to a strongly conserved epitope outside the receptor-binding motif (RBM); and class 4 antibodies (e.g. CR3022) bind to a cryptic epitope of the RBD and do not interfere with ACE2 binding (1–3).

Up to date, five SARS-CoV-2 variants of concern (VOCs) have emerged. While wild-type (WT) strains and VOC Alpha are susceptible to all monoclonal antibodies, VOCs Beta and Gamma have accumulated amino acid exchanges K417N, E484K, and N501Y, which impair the neutralizing activity of class 1 and 2 antibodies (4). Class 3 antibodies, however, are still active because they bind to a more conserved cross-neutralizing site (4, 5). VOC Delta is more infectious because it binds to low levels of ACE2 (6), while VOC Omicron is a true immune escape variant that is no longer neutralized by most monoclonal antibodies (7, 8).

*In vitro*, SARS-CoV-2 escape mutants occurred in the presence of single neutralizing class 1 or class 2 antibodies, but not with a non-competing antibody cocktail (9). We aimed to study the plasticity of the viral spike protein in the presence of class 3 antibody S309. This antibody was detected in a patient infected with SARS-CoV in 2003 and isolated in 2013 using a memory B cell screen in this patient (3). S309 binds a highly conserved epitope within the SARS-CoV-2 RBD with high affinity (1) and has the potential to broadly neutralize within the sarbecovirus subgenus, including SARS-CoV-2 (3). Notably, S309 retained its neutralizing activity against several SARS-CoV-2 VOCs *in vitro*, including Alpha, Beta, Gamma and Delta (10, 11). In May 2021, the stabilized version of S309 with enhanced Fc receptor binding, sotrovimab, received emergency approval as a therapeutic antibody to prevent disease progression in high-risk early-stage COVID-19 patients based on the results of a phase 3 study (12, 13).

While sotrovimab is still reasonably active against most viruses of Omicron sublineage BA.1 (10), neutralization efficacy decreased significantly with the emergence of sublineage BA.2 (14–16). As a result, the FDA revised the approval of the emergency use of sotrovimab for the treatment of COVID-19 in any U.S. region in April 2022 due to the high prevalence of BA.2 infections (17). Although evasion from S309 neutralization has been reported in the context of VOC Omicron sublineages, a recent study has demonstrated that protection was maintained for BA.1, BA.1.1 and BA.2 viruses in an animal model (18).

Here we describe the escape of SARS-CoV-2 from monoclonal antibody S309 in an *in vitro* viral evolution experiment and provide insights into the underlying escape mechanism, by investigating whether the evolution of resistance occurs concomitantly with a shift in the mode of viral entry.

## Materials and methods

### Cell culture

SARS-CoV-2 was propagated in Vero, HEK293T and CaCo-2 cells (CLS Cell Lines Service, Eppelheim, Germany) in DMEM (Gibco, Waltham, MA) supplemented with 10% fetal calf serum and 1% streptomycin/penicillin (Pan Biotech, Aidenbach, Germany). Cells were regularly tested for mycoplasma contamination and monitored daily for viability and cell density to provide optimal growth conditions. Vero cells were plated at least four hours before infection, CaCo-2 and HEK293T cells one day before infection. Cell culture experiments with replication-competent SARS-CoV-2 were performed under biosafety level 3 conditions and were conducted in accordance with all relevant local legislation.

### Selection of antibody-resistant SARS-CoV-2

Vero cells were infected with the “input virus” (GenBank accession no. ON715117) starting with a multiplicity of infection (MOI) of 0.05, as described previously (19). The input virus was derived from SARS-CoV-2 WT strain CA (GenBank accession no. MZ675816), which had developed a deletion of 9 amino acids (ΔI68-ΔT76) after serial passaging in Vero cells. Supernatants were transferred weekly to fourfold increased antibody concentrations (0.0625-64 µg/ml). Viral loads were quantified 5 days post infection (p.i.) using RT-qPCR after extraction of viral RNA from cell culture supernatants using DLR buffer (0.1 M NaCl, 0.01 M Tris, 0.5% IGEPAL CA-630 in DEPC H20, pH 7.4) mixed with RNAse inhibitor (Applied Biosystems, Darmstadt, Germany) (20). Equal volumes of DLR buffer and cell culture supernatants were incubated at room temperature for 30 min. SARS-CoV-2 RNA was reverse transcribed and amplified with Taq-Path-Mix (Metabion international, Planegg, Germany) using published primers and probes (21) on a StepOnePlus Real-time PCR system. Viral loads were quantified using an *in vitro* transcribed RNA, as described previously (22). Antibody resistance was assumed when viral loads at the highest antibody concentration (64 µg/ml) were comparable to the uninhibited virus control.

### Neutralization and inhibition assay

Vero, HEK293T, and CaCo-2 cells were seeded at a density of 15.000 cells/well in flat bottom 96-well plates. Serial fourfold dilutions of antibodies were pre-incubated with the different viral strains (MOI 0.05) for 1h at 37°C. Thereafter, the antibody-virus mixture was added to the cells. Cells without antibody and without virus served as ‘cell controls’, cells without antibody but with virus as ‘virus controls’ and cells fixed with 4% paraformaldehyde (Sigma-Aldrich, St. Louis, MO), washed with DPBS for five times, and infected in parallel as ‘background controls’. Control wells fixed with paraformaldehyde were plated separately to prevent evaporation and interference with non-fixed cell layers and viruses. At 2h p.i., cells were washed and antibodies were replenished, and at 48h p.i., supernatants were harvested and analyzed using SARS-CoV-2 RT-qPCR as described above. A similar set-up was used for testing the susceptibility of SARS-CoV-2 variants aloxistatin and camostat as inhibitors of endocytosis and fusion, respectively (both MedChemExpress, Monmouth Junction, NJ). Toxicity of both inhibitors at indicated concentrations was excluded using the 3-(4,5-dimethylthiazol-2-yl)-2,5-diphenyl¬tetrazolium bromide (MTT) assay (23). The percent neutralization was calculated as 100-(viral load of inhibited sample/viral load of uninhibited virus control)*100.

### Next generation sequencing

RNA was extracted from cell culture supernatants using the EZ1 Advanced XL platform (Qiagen, Hilden, Germany) and quantified using real-time PCR as described above. Whole genome sequencing of SARS-CoV-2 was performed by targeted PCR-based amplification using the Ion AmpliSeq™ SARS-CoV-2 Research Panel (Thermo Fisher Scientific, Waltham, USA). This panel targets 237 amplicons specific to SARS-CoV-2 covering >99% of the viral genome along with 5 human expression controls. Viral copy numbers were normalized by diluting total nucleic extracts to the lowest concentrated sample within one run comprising batches of 16 samples, followed by reverse transcription using the SuperScript™ VILO™ cDNA Synthesis Kit (Thermo Fisher Scientific). Sequencing libraries were automatically prepared using the IonChef™ instrument (Thermo Fisher Scientific). Amplification cycles were set depending on the viral load according to the manufacturer’s specifications. The final library pool was quantified by real-time PCR using the KAPA Library Quantification Kit on a LightCycler 480 II instrument (Roche Diagnostics, Mannheim, Germany) and subjected to high-throughput sequencing on the IonTorrent™ Genestudio S5 Plus instrument (Thermo Fisher Scientific). The Torrent Suite 5.12.2 was used for basecalling and demultiplexing. Processed reads were further analyzed with the SARS-CoV-2 Research Plug-in Package. Reads per genome were mapped to the SARS-CoV-2 MN908947 (Wuhan-Hu-1) reference genome. Samples with a mean genome coverage above 1,000-fold and frequency of ambiguous bases above 1% were eligible for calling of consensus sequences by IRMAreport v1.3.0.2. Single nucleotide polymorphisms were detected by variantCaller v5.12.0.4 and annotated with COVID19AnnotateSnpEff v1.3.0.2. Whole genome sequences of SARS-CoV-2 strains obtained at week 8 (R346S) and week 12 (7S1) of the S309 selection procedure have been deposited in GenBank (accession nos. ON003598 and ON003597, respectively). In addition, whole genome sequences of SARS-CoV-2 strains obtained at week 12 in the absence of antibodies (VC12) or presence of CR3022 (7C1) have been deposited in GenBank (accession nos. ON630347 and ON630346, respectively). All SARS-CoV-2 strains used in this study are assigned to Pango lineage B.1.1 (24).

### Generation of SARS-CoV-2 spike protein RBD variants, soluble ACE2, and monoclonal antibodies

Generation of the Wuhan-Hu-1 RBD-encoding plasmid and purification of antigens was performed as described previously (19). Plasmids encoding mutated RBDs were generated *via* insertion of P337L and/or R346S into the WT sequence *via* overlap extension PCR (25) and re-cloning into the original pcDNA5/FRT/TO-derivate. Expi293F™ cells (Thermo Fisher Scientific; A14527) were transfected with these plasmids as recommended by manufacturer. After 5d, supernatants were harvested by centrifugation and loaded onto immobilized metal chelate affinity chromatography (IMAC) columns (HisTrap Excel, Cytiva). After washing with DPBS containing 10 mM imidazole (Sigma), proteins were eluted over a linear 10–500 mM imidazole gradient in PBS. The protein buffer was exchanged to PBS and concentrated to approximately 1–2 mg/ml by ultrafiltration. The ACE2 construct (amino acid 20-732) was codon optimized and synthesized by GeneArt AG (Thermo Fisher Scientific) and cloned into a pcDNA5/FRT/TO derivate providing a mini-tPA-signal peptide (26) and an avi-his8-tag (sequence GS-GLNDIFEAQKIEWHE-GS-HHHHHHHH). Proteins expressed in Expi293F cells (see above) were purified by IMAC and subsequent anion exchange chromatography (HiTrap DEAE Sepharose, Cytiva) using a gradient from 10 mM to 1 M NaCl, in HEPES pH 6.8. Protein was buffer exchanged to PBS and stored at 4°C. Site-specific biotinylation was performed using BirA (BirA biotin-protein ligase standard reaction kit, Avidity).

Light chain and heavy chain variable domain sequences from the monoclonal antibodies were retrieved from NCBI GenBank or RSCB PDB (NCBI accession numbers DQ168569 and DQ168570 for CR3022; pdb code 6WPT for S309; pdb code 6XDG for REGN10933 and REGN10987). Sequences were optimized for human codon usage, synthesized by GeneArt AG and cloned into a pcDNA5/FRT/TO derivate providing a murine IgG1 signal peptide and the constant regions of lambda light chain (CR3022, S309, REGN10933) or kappa light chain (REGN10987) and human IgG1 heavy chain. Monoclonal antibodies were transiently expressed at a gene dosage ratio of 1 for light and heavy chain plasmids in Expi293F™ cells. Antibodies were purified from supernatants by protein A affinity chromatography (HiTrap MabSelect SuRe, Cytiva). IgGs were eluted by a pH step using 100 mM glycine buffer at pH 3.2 and the eluted antibodies were immediately buffer exchanged to PBS.

### Binding ELISAs

Binding of antibodies to the different RBDs was analyzed in duplicates using a similar ELISA format as recently described (19). Flat bottom 96-well plates (Thermo Fisher, Nunc Maxisorp, 44-2404-21) were coated with 2 µg/ml of the respective protein overnight. After blocking and washing, plates were incubated with eight four-fold serial dilutions of the antibodies or soluble and biotinylated ACE2 starting at 80 nM. After incubation with the anti-human IgG-HRP conjugate or Streptavidin-HRP conjugate (Roche), TMB substrate solution (Mikrogen) was added and plates were developed for 4 min. Optical density at 450 nm and 600 nm was measured immediately. Values were curve fitted using 4-parameter logistic regression (4PL, GraphPad Prism version 9.2.0) after background subtraction. Endpoint titers were calculated from the titration data in combination with the previously determined cut-off values.

### Grating-coupled interferometry measurements of RBD-antibody interactions

To determine dissociation constant (KD) and rate constants (kon and koff) of the binding of ACE2 and S309 to the investigated RBD variants, Grating-Coupled Interferometry (GCI) was measured on a WAVEsystem (Creoptix) using the waveRAPID method. Details of the experimental settings of S309 binding to RBD variants are given in **Supplementary Table 1**. ACE2 was biotinylated prior to the GCI analysis as described above. Thereupon, it was bound *via* streptavidin to the sensor chip. Experimental parameters of the ACE2 interaction with RBD variants are given in **Supplementary Table 2**.

### Visualization of protein structures

Protein structures were visualized using Pymol (version 2.5.2, LLC Schrodinger). Structural data were obtained from the Protein Data Bank (PDB) (https://www.rcsb.org). Data files used in this study are available at entry codes 6WPS (3) and 6M17 (27).

### Statistics

Three or more groups were compared using repeated measures (RM) one-way ANOVA, adjusted for multiple testing using Tukey’s or Dunnett’s correction. Two-sided p values <0.05 were considered significant.

## Results

### Selection of an antibody escape variant of SARS-CoV-2

**Figure 1:**
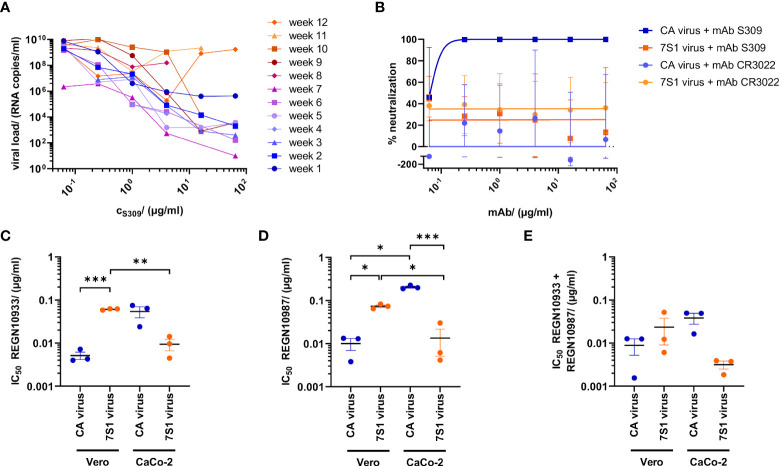
Selection and characterization of antibody-resistant SARS-CoV-2. **(A)** Cultivation of the SARS-CoV-2 input strain (GenBank accession no. ON715117) in Vero cells for 12 weeks, using increasing concentrations (c) of neutralizing monoclonal antibody (mAB) S309 or non-neutralizing mAB CR3022 (see **Supplementary Figure 1**). Data show mean viral loads in cell culture supernatants of each passage at 5d post infection, transferred weekly in duplicates to fresh cells containing fourfold increased antibody concentrations. A virus control without antibodies was included in each passage and continuously propagated during the entire selection period. **(B)** Neutralization of input strain (CA) and resistant (7S1) virus by increasing S309 and CR3022 concentrations. **(C–E)** Neutralizing activity (inhibitory concentration 50%, IC50) of **(C)** REGN10933, **(D)** REGN10987 and **(E)** a combination of both Regeneron antibodies on Vero and CaCo-2 cells. Viral loads were determined in cell culture supernatants using RT-qPCR 2d post infection and plotted as percent neutralization in a non-linear fit. Data show mean and standard error of three independent experiments. Statistics is based on repeated measures (RM) one-way ANOVA, adjusted for multiple testing using Tukey’s correction. *p<0.05; **p<0.01; ***p<0.001.

To obtain a SARS-CoV-2 variant with escape to neutralizing class 3 antibody S309, WT strain CA was passaged weekly in Vero cells in the presence of increasing concentrations of this antibody (0.0625-64 µg/ml). S309 was originally discovered in SARS-CoV infection and recognizes a highly conserved proteoglycan epitope in the sarbecovirus spike protein distinct from the RBM (1, 3). The input virus was neutralized by S309 with an IC50 of 0.09 µg/ml (600 pM), which is very similar to recently published data (10). After twelve rounds of selection, viral replication was no longer suppressed by the highest antibody concentration (64 µg/ml) (**Figure 1A**). In contrast to input virus CA, the viral stock of the resistant strain (7S1), obtained at the end of the selection process, was no longer susceptible to S309 inhibition (**Figure 1B**). Parallel propagation of the input virus with CR3022, a non-neutralizing class 4 antibody, did not inhibit virus replication during the entire cultivation period (**Supplementary Figure 1**). Altogether, SARS-CoV-2 developed complete resistance against S309 within three months of continuous selection.

### Reduced susceptibility to monoclonal antibodies targeting the RBM

S309 contacts with its long heavy chain complementarity-determining region (CDR) 3 residues 337-344 of the N-terminal alpha helix in the RBD. With its light chain CDR 2, it further interacts with the conserved N-glycan at position 343 (3). Light chain CDR 1 and CDR 2 extend the paratope of S309 by interacting with residues 440-444. The epitope of S309 is thus located outside the RBM, which is the target of class 1 and 2 antibodies (3). To further characterize the effects of S309 resistance, we analyzed the inhibitory effect of the two well-described antibodies REGN10987 (imdevimab) and REGN10933 (casirivimab), both of which bind to different, non-overlapping RBM epitopes (28). Using Vero cells, the S309-resistant strain was significantly less susceptible to REGN10987 (**Figure 1C**) and REGN10933 (**Figure 1D**) than the input virus (p<0.05), whereas the opposite effect was observed when CaCo-2 cells were infected. This difference decreased with the application of the antibody cocktail (**Figure 1E**). By developing resistance to S309, which binds outside the ACE2 RBM, SARS-CoV-2 changed its susceptibility to two antibodies that compete with ACE2 binding.

### Targeted escape of SARS-CoV-2 spike protein upon S309 selection

**Figure 2:**
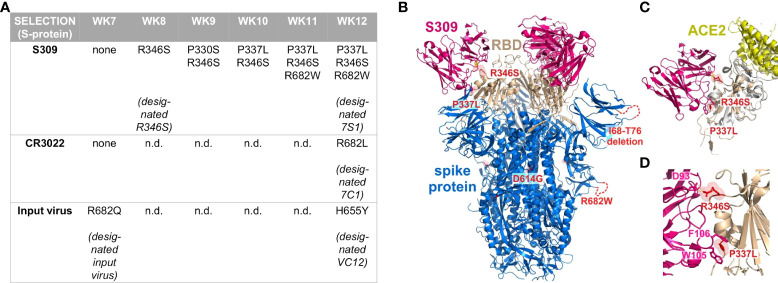
Characterization of *de novo* SARS-CoV-2 spike amino acid exchanges in the course of S309 selection. **(A)** Next generation sequencing of cell culture supernatants from weeks (WK) 7-12 of the antibody selection. The table displays spike protein amino acid exchanges that emerged over time, using the sequence of the input virus as reference (GenBank accession no. ON715117). Sequences of 7S1 (ON003597), 7C1 (ON630346), and VC12 (ON630347) were also deposited at GenBank. **(B)** Projection of *de novo* amino acid exchanges R346S, P337L, and R682W onto the SARS-CoV-2 spike protein trimer (blue) in complex with the neutralizing antibody S309 (pink) at 3.10 Å resolution. The dashed lines show a non-resolved amino acid stretch of the SARS-CoV-2 spike protein including a deletion at positions 68 to 76 (ΔI68-ΔT76), which was already present in the input virus **(C, D).** Close-up views of amino acid exchanges R346S and P337L, which did not interfere with ACE2 (yellow) binding to the receptor-binding domain (wheat), but impaired the interaction of the receptor-binding domain with S309. Structures were visualized based on Protein Data Bank entries 6WPS and 6M17. Protein structures were visualized using Pymol (version 2.5.2, LLC Schrodinger).

To determine how SARS-CoV-2 responded to S309 selection at a genomic level, we performed whole genome sequencing for passages 7-12 of our long-term culture. In the spike protein, a large deletion of 9 amino acids (ΔI68-ΔT76) was present in the input virus and persisted in all strains during propagation. During selection with the monoclonal antibodies, amino acid exchanges R682W and R682L were detected at weeks 11 (S309) and 12 (CR3022), respectively (**Figure 2A**). When the input virus was passaged in the absence of monoclonal antibodies, amino acid exchange R682Q appeared after 7 weeks. The amino acid exchanges at position 682 are therefore most likely an adaptation to cultivation in Vero cells. After 12 weeks of propagation, the input virus aquired H655Y, which was associated with increased spike cleavage and replication *in vitro* (29). To exclude effects caused by H655Y, we used the input virus as a control for further functional characterization of selected SARS-CoV-2 strains. Under selection with S309, R346S appeared and persisted from week 8 until the end of the observation period (**Figure 2A**). This amino acid exchange is part of the S309 epitope and also important for REGN10987 binding (1, 30). At week 9, amino acid exchange P330S occurred, but was quickly displaced by viral quasispecies harboring P337L. Overall, within three months of selection with S309, SARS-CoV-2 accumulated two amino acid exchanges (R346S, P337L) predicted by structural modeling to be part of this monoclonal antibody’s epitope and located in the N-terminal helix of the RBD (**Figures 2B–D**).

### Reduced binding of S309 to RBD expressing P337L

**Figure 3:**
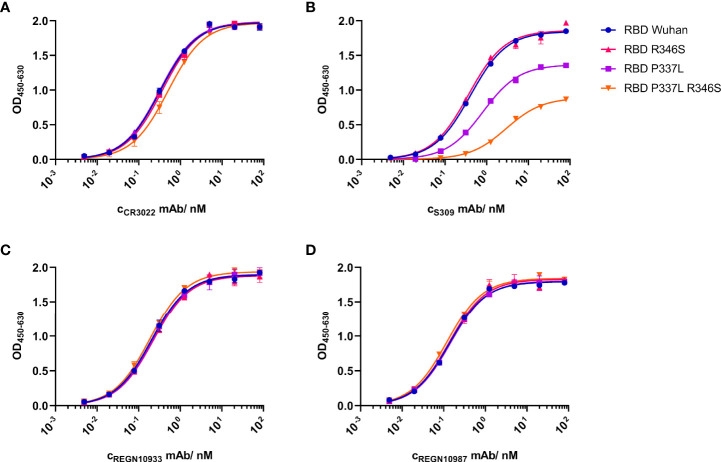
Impact of *de novo* amino acid exchanges R346S and P337L on the binding of monoclonal antibodies to the SARS-CoV-2 receptor-binding domain (RBD). Recombinant soluble variants of RBDs with the Wuhan-Hu-1 sequence (blue circles), R346S (red triangle), P337L (green square), or both amino acid exchanges (orange triangle) were analyzed in an ELISA. Binding affinities of monoclonal antibodies (mAB) were measured as optical density (OD) across eight serial dilutions (80 nM to 49 pM) of **(A)** non-neutralizing CR3022, **(B)** neutralizing S309, and neutralizing **(C)** REGN10933 and **(D)** REGN10987 antibodies. Data are shown as mean of duplicates out of one experiment. Corresponding Grated-Coupled Interferometry data are shown in **Supplementary Figure 2**.

In a next step, we wanted to evaluate the contribution of amino acid exchanges R346S and P337L to the resistant phenotype. R346 is one of four residues (N343, R346, N440, L441) within the S309 epitope (1). Regarding P337, Starr et al. reported that any substitution of the helix breaker proline at this position resulted in a complete S309 escape (31). Notably, amino acid exchanges at this position were also observed in patients treated with the S309-derived antibody sotrovimab in the COMET-ICE trial (32). To determine the influence of each amino acid substitution on the affinity to the antibodies used in this study, we expressed RBDs with either one or both amino acid exchanges (R346S, P337L) and analyzed their binding to S309, CR3022, REGN10933 and REGN10987 in comparison to the Wuhan-Hu-1 RBD that resembled the original clinical isolate used for selection. In an ELISA, CR3022 bound all RBDs similarly well (**Figure 3A**), while S309 bound RBDs Wuhan-Hu-1 and R346S more efficiently than RBD P337L and in particular RBD P337L+R346S (**Figure 3B**). Thus, the first variant to appear in the selection (R346S) was still bound by S309, while P337L and especially the double mutant clearly escaped recognition by this antibody. Although position 346 is part of the REGN10987 epitope, RBD variants were bound by both REGN antibodies similarly well (**Figures 3C, D**). The altered susceptibility of the S309-resistant SARS-CoV-2 to the REGN antibodies was thus not explained by reduced RBD binding as shown by ELISA.

### Preserved binding of ACE2 to mutated RBDs

We hypothesised that R346S increases the affinity of RBD for ACE2 and thus SARS-CoV-2 infectivity. First, we investigated binding of WT as well as single and double RBD variants to S309 using Grating-Coupled Interferometry (GCI). As in the ELISA, WT RBD (KD=555 pM) and R346S (KD=401 pM) showed a clear dose-responsive binding with surface saturation, while RBD P337L and the double mutant RBD P337L+R346S did not bind (**Supplementary Figures 2A–D**). Binding of RBDs to ACE2 was analyzed using a C-terminally biotin-tagged soluble version (**Supplementary Figures 2E–H**). Differences between the KD values were small, with RBD WT (10 nM) and RBD R346S (11 nM) binding slightly more efficiently to ACE2 than RBD P337L (15 nM) and RBD P337L+R346S (20 nM). As expected, such subtle differences could not be resolved by ELISA (**Supplementary Figure 2I**). As a control, we studied oligomerization and homogeneity of the proteins in a size exclusion chromatography experiment. All proteins showed a symmetric peak at comparable retention times corresponding to a monomeric oligomerization state, were homogeneous and displayed comparable hydrodynamic properties (**Supplementary Figure 2J**). According to the GCI experiments, amino acid exchange P337L reduced RBD affinity to S309, whereas the effect of all amino acid exchanges on the binding of ACE2 was small.

### High infectivity of resistant SARS-CoV-2 strain for human cells

**Figure 4:**
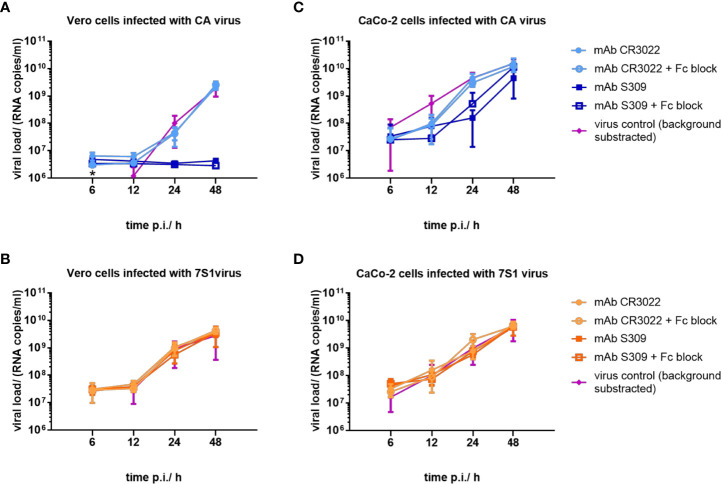
Replication kinetics of SARS-CoV-2 strains before and after selection with S309. **(A, B)** Vero and **(C, D)** CaCo-2 cells were infected with input (CA) and resistant (7S1) SARS-CoV-2 viruses using a multiplicity of infection of 0.05. Viruses were cultivated with CR3022 (squares) and S309 (circles) at a concentration of 5 µg/ml in the absence and presence of FcR-blocking reagent. Viral supernatants were harvested at 6h, 12h, 24h, and 48h post infection and analyzed using RT-qPCR. A control with paraformaldehyde-fixed cells was included to quantify background viral load. Virus control (triangles) shows viral loads in the absence of antibodies with background subtracted. Mean and standard error of three independent experiments on Vero cells and four independent experiments on CaCo-2 cells are shown. The asterisk in **Figure 4A** indicates a mean viral load in the virus control below the background level at 6h p.i.

In a next step, we compared the infectivity of input and selected SARS-CoV-2 strains using African green monkey (Vero) and human (CaCo-2) cell lines. In Vero cells, S309 inhibited input virus infection (**Figure 4A**), but not infection with the resistant viral strain (**Figure 4B**), independent of Fc receptor blocking. S309 reduced input virus infection to a lesser extent in CaCo-2 cells compared to Vero cells (**Figure 4C**), as recently described for non-RBM antibodies in ACE2-overexpressing cells (33). As expected, S309 did not block CaCo-2 cell infection with the resistant viral strain; again, Fc receptors were not involved (**Figure 4D**). Altogether, the resistant strain showed a similar fitness compared to the input virus in both African monkey and human cells.

### Shifting the mode of cell entry from endocytosis to fusion

Next, we wanted to investigate whether the two amino acid exchanges alter the entry of SARS-CoV-2 into target cells. The mode of entry is largely determined by the expression of TMPRSS2: cells with low expression of TMPRSS2 but high expression of Cathepsin-L (e.g. Vero cells) support SARS-CoV-2 entry mainly by endocytosis, while cells with high levels of TMPRSS2 and low levels of Cathepsin-L (e.g. CaCo-2 cells) support fusion (34). HEK293T cells lacking TMPRSS2 behave like Vero cells and exclusively support endocytosis (35, 36). We further analyzed the mode of entry of input and resistant strains using camostat and aloxistatin as inhibitors of fusion and endocytosis, respectively (37).

**Figure 5:**
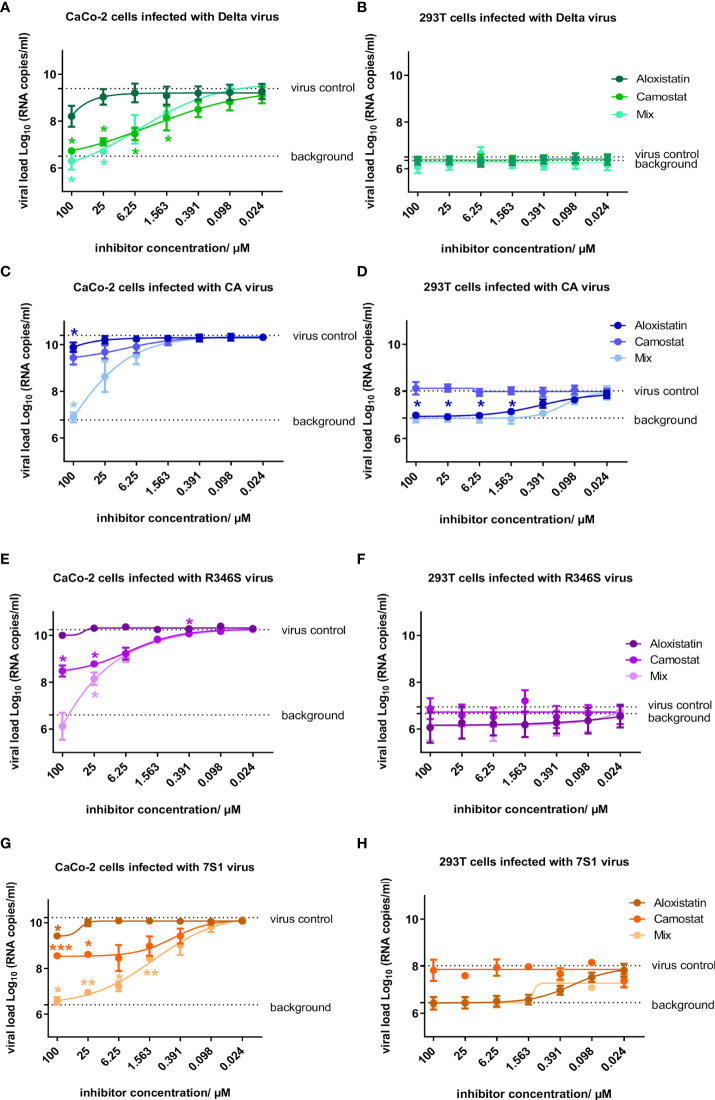
Susceptibility of SARS-CoV-2 variants to inhibition of endocytosis and fusion by aloxistatin and camostat, respectively. CaCo-2 and HEK293T cells were infected with **(A, B)** a SARS-CoV-2 Delta strain (39), **(C, D)** wild-type virus (CA) used as input for selection of S309-resistant strains, and mutant strains with **(E, F)** R346S and **(G, H)** R346S+P337L (7S1), using a multiplicity of infection of 0.05. Cells were propagated in the presence of the endocytosis inhibitor aloxistatin, the fusion inhibitor camostat or both in a 1:1 ratio (mix) at concentrations of the individual inhibitors between 0.024-100 µM. At 2d post infection, SARS-CoV-2 concentrations were determined in cell culture supernatants and plotted as log viral load in a non-linear fit, using cells infected in the absence of inhibitors (virus control) and cells fixed with 4% paraformaldehyde (background control) as constraints. Data show mean and standard error of three independent experiments. Statistics was calculated using one-way ANOVA with Dunnett’s correction, comparing each inhibitor concentration with the respective uninhibited control (*p<0.05, **p<0.01, ***p<0.001). Macromolecular electrostatics of the SARS-CoV-2 receptor-binding domain (RBD) upon evolution of amino acid exchange R346S is shown in **Supplementary Figure 3**.

The well-characterized VOC Delta, which enters cells mainly by fusion (38), was inhibited by camostat in CaCo-2 cells, while HEK293T cells were not infected (**Figures 5A, B**). The opposite phenotype was observed in the input strain, which was inhibited by aloxistatin in HEK293T cells, while camostat showed only little effect in CaCo-2 cells (**Figures 5C, D**). The R346S strain showed a Delta-like phenotype with preferential inhibition by camostat and no replication in HEK293T cells (**Figures 5E, F** **)**. The strain carrying R346S+P337L (7S1) was also inhibited by camostat, but apparently regained the ability to infect *via* endocytosis, as replication was observed in HEK293T cells (**Figures 5G, H** **)**. Thus, the input virus enters the cells mainly *via* endocytosis, while the single mutant virus shifted to fusion and the double mutant served both entry mechanisms equally well.

## Discussion

Within 12 weeks of cell culture, SARS-CoV-2 developed complete resistance to S309, an antibody targeting a conserved epitope of SARS-CoV and SARS-CoV-2. Our data show that SARS-CoV-2 can develop an escape mutant in a conserved area of the viral spike protein and still remain viable and infectious, as shown in African green monkey and human cell lines. This process took some time, while resistance against antibodies targeting the RBM appears to develop much faster (9, 30, 40). A direct comparison was not possible, because we did not select SARS-CoV-2 in parallel with REGN antibodies. The S309-resistant virus also showed reduced susceptibility to REGN10987 and REGN10933 in Vero cells, which is in line with data that P337L reduces the susceptibility to REGN10987 fivefold (41). The impaired susceptibility to REGN10987 may be also explained by the partially overlapping epitope with S309 at position 346 (1). However, a more general effect on the viral spike protein may apply, because the susceptibility to REGN10933 was also affected, although the epitopes do not overlap.

Amino acid exchanges E340A/K/G/Q and P337L/R/K were reported to be accompanied by a pronounced increase in IC50 against S309 in WT SARS-CoV-2 (10) as well as in VOC Delta (42, 43) and Omicron (44, 45). In our S309 selection experiment, SARS-CoV-2 first developed amino acid exchange R346S, which was described as ‘immune-escape enabling’ amino acid exchange for S309 (46). Together with residue N440, position 346 was described to be part of the epitope of S309 and important for binding of S309 (1). R346S alone was not sufficient to alter S309 binding, as seen in our ELISA and GCI experiments. However, R346S in combination with P337L enhanced resistance to S309 in our experiments. In this respect, it is noteworthy that amino acid exchange R346K in VOC Omicron BA.1.1 reduced sensitivity to sotrovimab (14).

Amino acid exchange P330S, which was also detected in an immunocompromised patient on day 93 during long-term infection with COVID-19 (47), appeared only briefly at week 9 of the selection process, but was quickly replaced by P337L. Significant resistance only emerged with amino acid exchange P337L, as it abolished binding to S309, especially in combination with R346S. Our results obtained by ELISA and Grating-Coupled Interferometry corroborate the data of Starr et al. according to which the loss of proline at position 337 leads to a complete escape from binding of the RBD to S309 (48). Notably, the amino acid changes R346S and P337L occurred in 3774 and 328 sequences deposited into GISAID database, respectively (49).

The entry of SARS-CoV-2 into target cells is a multi-step process involving spike protein rearrangements upon binding to ACE2. After translation, the spike protein is cleaved into subunits S1 and S2 by furin-like proteases in the Golgi apparatus of the virus-producing cell; S2 is further cleaved by transmembrane serine protease 2 (TMPRSS2) and cathepsin L at the surface and in the endosomal compartment of the newly infected cells, respectively (50). An intact furin cleavage site is important for efficient replication in human cells (51). Propagation of SARS-CoV-2 in Vero cells frequently leads to loss of the polybasic cleavage site (RRAR, amino acids 682-685) at the S1-S2 junction (52–54), limiting the range of cell tropism and the ability to utilize the TMPRSS2 pathway (55). We observed three different amino acid exchanges at position 682 within the polybasic cleavage site that occurred late during selection with S309 and CR3022 as well as in the absence of antibodies (R682W, R682L and R682Q, respectively), suggesting an adaptation to cultivation in Vero cells. In a similar selection experiment, R682W was identified in addition to a five amino acid deletion (Δ675-Δ679) in close proximity of the furin cleavage site (10).

Sotrovimab is a stabilized version of S309, whose half-life was extended by a modification of the crystallizable fragment (Fc) (10). It neutralizes SARS-CoV-2 VOCs Alpha (B.1.1.7), Beta (B. 1.351), Gamma (P.1) and Delta (B.1.617.2) in live- and pseudovirus systems, and also retains neutralizing activity against Omicron BA.1 (10, 56). In addition, it remains active against variants carrying resistance-associated amino acid exchanges to currently authorized monoclonal antibodies (10). In a multicenter, double-blind phase 3 study, sotrovimab reduced disease progression and hospitalization by 85% in high-risk outpatients with mild-to-moderate COVID-19 (12). In the COMET-ICE trial, a number of amino acid exchanges occurred *in vivo*, of which P337L, E340A and E340K were shown to confer resistance to S309 in a pseudovirus system (32). In our *in vitro* experiment, amino acid exchange P337L was selected, suggesting that some conclusions can be drawn about the situation *in vivo*. Our data confirm a high resistance barrier of S309, but also show that resistant and highly infectious escape variants can arise with a low number of targeted amino acid exchanges. This finding could be of particular importance for immunocompromised patients in whom SARS-CoV-2 replicates over an extended period of time (47). Here, sotrovimab may be used in combination with other antibodies that interfere with ACE-2 binding (1).

Notably, SARS-CoV-2 responded to neutralization by S309 with an altered mode of cell entry. The observed adaptation is consistent with viral evolution under selection pressure, as S309 preferentially blocked endocytotic uptake of SARS-CoV-2 in HEK293T and Vero cells, which express no or only low levels of TMPRSS2 (34, 35), respectively (**Figure 4** and **Supplementary Figure 4**). In contrast, S309 did not efficiently block SARS-CoV-2 entry into CaCo-2 cells expressing TMPRSS2 for fusion (**Figure 4**). Thus, S309 triggered a shift in cell entry in favor of membrane fusion as part of the antibody selection process and antibody escape. Here, amino acid exchange R346S changed the tropism from preferential endocytosis to fusion. Replacement of an arginine with serine results in the loss of a basic residue and reduces the overall size of the positively charged patch dominated by amino acids R346, R355, K444, and R466 (**Supplementary Figure 3**). This patch has been reported to interact with heparan sulfate (57), suggesting that binding of SARS-CoV-2 to this surface molecule promotes cell entry by endocytosis. The additional amino acid exchange P337L apparently allowed SARS-CoV-2 to infect HEK293T cells again, so that endocytosis and fusion could be used equally well for cell entry. Amino acid exchanges that occur in the context of S309 resistance could therefore trigger a more fusogenic remodeling of the viral spike protein (58). This remodeling could also affect the inhibitory effect of the Regeneron antibodies, which differentially suppressed the input and resistant virus on Vero and CaCo-2 cells.

Several mechanisms such as steric hindrance, S-glycoprotein cross-linking, or aggregation of virions (3, 59) are suspected to mediate the neutralization effect of S309. Therefore, it would be of interest to study the structural and functional consequences of the R346S and P337L amino acid exchanges *via* cryo-electron microscopy of the trimer variants and the resulting viral size distribution upon S309 treatment e.g. by dynamic light scattering measurements. A conformational change could also explain the different effects of REGN antibodies on input and resistant viruses depending on whether CaCo-2 or Vero cells were infected (**Figures 1C–E**). The S309-resistant strain carried additional amino acid exchanges in the multispecific protease nsp3 (D663A) and in the helicase nsp13 (P47L), the latter detected in naturally occurring SARS-CoV-2 isolates (60). Both proteins are involved in SARS-CoV-2 replication and may contribute to increased replication capacity of the S309-resistant strain. Altogether, the development of S309 resistance could increase infectivity and pathogenicity of SARS-CoV-2 (38). These data can be particularly valuable in times of emerging global VOC Omicron sublineages and beyond (14).

## Data availability statement

The datasets presented in this study can be found in online repositories. The names of the repository/repositories and accession number(s) can be found below: GenBank, under accession numbers: ON715117, MZ675816, ON003598, ON003597, ON630347 and ON630346.

## Author contributions

Conceptualization, DP, BS; Methodology, CM, AH, PS, AR, DP; Investigation, CM, AH, DP; Writing – Original Draft, BS; Writing – Review & Editing, CM, AH, PS, JM, DP, BS; Funding acquisition, AG, PS, BS; Resources, AG; Supervision, PS, JM, AG, DP, BS. All authors contributed to the article and approved the submitted version.

## Funding

This study was supported through the pandemic responsiveness fund of The Bavarian Ministry of Science and Art.

## Acknowledgments

We thank Benjamin Zimmer (2bind, Regensburg) for the excellent management of the Grating-Coupled Interferometry and Sakhila Ghimire for kindly providing CaCo-2 cells.

## Conflict of interest

The authors declare that the research was conducted in the absence of any commercial or financial relationships that could be construed as a potential conflict of interest.

## Publisher’s note

All claims expressed in this article are solely those of the authors and do not necessarily represent those of their affiliated organizations, or those of the publisher, the editors and the reviewers. Any product that may be evaluated in this article, or claim that may be made by its manufacturer, is not guaranteed or endorsed by the publisher.

## Supplementary material

Supplementary Table 1 Experimental settings of S309 binding to RBD variants via GCI.

Supplementary Table 2 Experimental settings of ACE-2 interaction with RBD variants via GCI

Supplementary Figure 1 Propagation of SARS-CoV-2 in the presence of non-neutralizing antibody CR3022. Cultivation of SARS-CoV-2 wild-type strain CA (GenBank accession no. ON715117) in Vero cells for 12 weeks, using increasing CR3022 concentrations (C). Data show viral loads in cell culture supernatants of each passage at 5d post infection, which were transferred weekly to fresh cells containing fourfold increased antibody concentrations.

Supplementary Figure 2 Grating-Coupled Interferometry (GCI) sensorgrams of the interaction between four receptor-binding domain (RBD) variants and S309 or ACE2. Response for one of two measured ligand channels is shown. Signals from the measurements are shown in red, curve fits with a 1:1 interaction model are shown in black. **(A)** Interaction between S309 and wild-type (WT) RBD. Dose-responsive binding with surface saturation was detected. Fitting the data from both ligand channels resulted in a calculated affinity of 555±192 pM. The surface activity was calculated to be 100%. **(B)** Interaction between S309 and RBD P337L. Binding of RBD P337L to the antibody was detected but no trend for surface saturation was observed. A complex binding behavior can be observed. Responses during the dissociation phase appear bi-phasic. Initially, responses decrease fast and then turn to remain on a certain level. Data cannot be described by a 1:1 interaction model. **(C)** Interaction between S309 and RBD R346S. Dose-responsive binding with surface saturation was detected. Fitting the data from both ligand channels measured resulted in a calculated affinity of 401±20 pM. The surface activity was calculated to be 100%. **(D)** Interaction between ACE2 and RBD P337L R346S. Responses are very weak but some minimal binding of this RBD to the antibody can be observed. Fitting of the data was not possible. **(E)** Interaction between ACE2 and WT RBD. Dose-responsive binding with surface saturation was detected. Fitting the data from both ligand channels measured resulted in a calculated affinity of 10±0.3 nM. The surface activity was calculated to be 100%. **(F)** Interaction between ACE2 and RBD P337L. Dose-responsive binding with surface saturation was detected. Fitting the data resulted in a calculated affinity of 15±0.3 nM. The surface activity was calculated to be 100%. **(G)** Interaction between ACE2 and RBD R346S. Dose-responsive binding with surface saturation was detected. Fitting the data resulted in a calculated affinity of 11±0.3 nM. The surface activity was calculated to be 100%. **(H)** Interaction between ACE2 and RBD P337L R346S. Dose-responsive binding with surface saturation was detected. Fitting the data resulted in a calculated affinity of 23±0.8 nM. The surface activity was calculated to be 100%. **(I)** ACE2 binding to four different RBDs immobilized on an ELISA plate using C-terminal site-specifically biotinylated ACE2 (ACE2-Biotin), titrated in fourfold serial dilutions starting at a concentration of 80 nM (n=3). Bound ACE2 was detected by a streptavidin-HRP-conjugate and subsequently quantified by a colorimetric reaction. **(J)** Size exclusion chromatography experiment showing that all RBD proteins adopt a monomeric oligomerization state, are homogeneous and display comparable hydrodynamic properties.

Supplementary Figure 3 Macromolecular electrostatics of the SARS-CoV-2 receptor-binding domain (RBD) upon evolution of mutations R346S and P337L. **(A)** Location of the RBD (cyan) and R346 (red) within the SARS-CoV-2 ectodomain. **(B)** Insight showing R346 within the extended RBD (adjacent domain shown in grey). **(C)** Surface potential of the extended RBD of wild-type and R346S mutant with the positively charged (blue) patch suspected to interact with heparansulfate. The patch (bordered by a dashed line) is disrupted in the R346S mutant. Electrostatic surface potential was calculated using the Adaptive Poisson-Boltzmann Solver plugin for PyMOL and (1).

Supplementary Figure 4 Susceptibility of SARS-CoV-2 strains to inhibition by monoclonal antibodies in HEK293T cells. Replication of SARS-CoV-2 input virus (CA, left panel) and S309-resistant strain (7S1, right panel) in HEK293T cells in the presence of CR3022 and S309 at a concentration of 5 µg/ml. Controls included HEK293T cells infected in the absence of monoclonal antibodies (virus control) and paraformaldehyde (PFA)-fixed HEK293T cells to quantify background viral load. Data show mean and standard deviation of one experiment performed in triplicates.
