# Supplementary material for: Targeted escape of SARS-CoV-2 in vitro from monoclonal antibody S309, the precursor of sotrovimab
Source: Front Immunol. 2022 Aug 24;13:966236. doi: 10.3389/fimmu.2022.966236 (PMC9449809; doi:10.3389/fimmu.2022.966236)
Supplement: Supplementary Table 1 — Experimental settings of S309 binding to RBD variants via GCI. [file DataSheet_1.docx]

Supplementary Material

# Supplementary Tables

## Suppl. Table 1: Experimental settings of S309 binding to RBD variants via GCI

| **Parameter** | **Variables, details and description** |
| --- | --- |
| Sensor chip | PCP-PAG |
| Ligand | Protein A/G captured S309 (captured to 50 pg/mm^2^ at a concentration of 500 ng/ml for each analysis cycle) |
| Ligand regeneration | Three 60 sec pulses with 10 mM Glycine-HCl pH 1.7, performed after each analysis cycle |
| Analytes | RBD variants at a concentration of 1 µM in running buffer |
| Running buffer | 10 mM HEPES pH 7.4, 150 mM NaCl, 3 mM EDTA, 0.05% Tween-20 |
| Association time | 150 sec, pulsed |
| Dissociation time | 900 sec |
| Flowrate | 50 µl/min |
| Acquisition rate | 1 Hz |
| Temperature | 25°C |
| Blank injections | Regularly interspaced every 4^th^ sample |
| DMSO calibration | 0.5% calibration solution, regularly interspaced every 5^th^ injection and at the beginning/end of the experiment |
| Data processing | Double-referencing |
| Data fitting | 1:1 kinetic model |

## Suppl. Table 2: Experimental settings of ACE-2 interaction with RBD variants via GCI

| **Parameter** | **Variables, details and description** |
| --- | --- |
| Sensor chip | PCP-STA |
| Ligand | Streptavidin-captured ACE2 (≈75 pg/mm^2^) |
| Analytes | RBD variants at a concentration of 500 nM in running buffer |
| Running buffer | 10 mM HEPES pH 7.4, 150 mM NaCl, 3 mM EDTA, 0.05% Tween-20 |
| Association time | 60 sec, pulsed |
| Dissociation time | 900 sec |
| Flowrate | 50 µl/min |
| Acquisition rate | 10 Hz |
| Temperature | 25°C |
| Blank injections | Regularly interspaced every 4^th^ sample |
| DMSO calibration | 0.5% calibration solution, regularly interspaced every 5^th^ injection and at the beginning/end of the experiment |
| Data processing | Double-referencing |
| Data fitting | 1:1 kinetic model |

# Supplementary Figures

**Supplementary Figure 1.** **Propagation of SARS-CoV-2 in the presence of non-neutralizing antibody CR3022.** Cultivation of SARS-CoV-2 wild-type strain CA (GenBank accession no. MZ675816) in Vero cells for 12 weeks, using increasing CR3022 concentrations (C). Data show viral loads in cell culture supernatants of each passage at 5d post infection, which were transferred weekly to fresh cells containing fourfold increased antibody concentrations.

**Supplementary Figure 2. Grating-Coupled Interferometry (GCI) sensorgrams of the interaction between four receptor-binding domain (RBD) variants and S309 or ACE2.** Response for one of two measured ligand channels is shown. Signals from the measurements are shown in red, curve fits with a 1:1 interaction model are shown in black. (**A**) Interaction between S309 and wild-type (WT) RBD. Dose-responsive binding with surface saturation was detected. Fitting the data from both ligand channels resulted in a calculated affinity of 555±192 pM. The surface activity was calculated to be 100%. (**B**) Interaction between S309 and RBD P337L. Binding of RBD P337L to the antibody was detected but no trend for surface saturation was observed. A complex binding behavior can be observed. Responses during the dissociation phase appear bi-phasic. Initially, responses decrease fast and then turn to remain on a certain level. Data cannot be described by a 1:1 interaction model. (**C**) Interaction between S309 and RBD R346S. Dose-responsive binding with surface saturation was detected. Fitting the data from both ligand channels measured resulted in a calculated affinity 401±20 pM. The surface activity was calculated to be 100%. (**D**) Interaction between ACE2 and RBD P337L R346S. Responses are very weak but some minimal binding of this RBD to the antibody can be observed. Fitting of the data was not possible. (**E**) Interaction between ACE2 and WT RBD. Dose-responsive binding with surface saturation was detected. Fitting the data from both ligand channels measured resulted in a calculated affinity of 10±0.3 nM. The surface activity was calculated to be 100%. (**F**) Interaction between ACE2 and RBD P337L. Dose-responsive binding with surface saturation was detected. Fitting the data resulted in a calculated affinity of 15±0.3 nM. The surface activity was calculated to be 100%. (**G**) Interaction between ACE2 and RBD R346S. Dose-responsive binding with surface saturation was detected. Fitting the data resulted in a calculated affinity of 11±0.3 nM. The surface activity was calculated to be 100%. (**H**) Interaction between ACE2 and RBD P337L R346S. Dose-responsive binding with surface saturation was detected. Fitting the data resulted in a calculated affinity of 23±0.8 nM. The surface activity was calculated to be 100%. (**I**) ACE2 binding to four different RBDs immobilized on an ELISA plate using C-terminal site-specifically biotinylated ACE2 (ACE2-Biotin), titrated in fourfold serial dilutions starting at a concentration of 80 nM (n=3). Bound ACE2 was detected by a streptavidin-HRP-conjugate and subsequently quantified by a colorimetric reaction. (**J**) Size exclusion chromatography experiment showing that all RBD proteins adopt a monomeric oligomerization state, are homogeneous and display comparable hydrodynamic properties.

**Supplementary Figure 3.** **Macromolecular electrostatics of the SARS-CoV-2 receptor-binding domain (RBD) upon evolution of mutations R346S and P337L.** (**A**) Location of the RBD (cyan) and R346 (red) within the SARS-CoV-2 ectodomain. (**B**) Insight showing R346 within the extended RBD (adjacent domain shown in grey). (**C**) Surface potential of the extended RBD of wild-type and R346S mutant with the positively charged (blue) patch suspected to interact with heparansulfate. The patch (bordered by a dashed line) is disrupted in the R346S mutant. Electrostatic surface potential was calculated using the Adaptive Poisson-Boltzmann Solver plugin for PyMOL and (1).


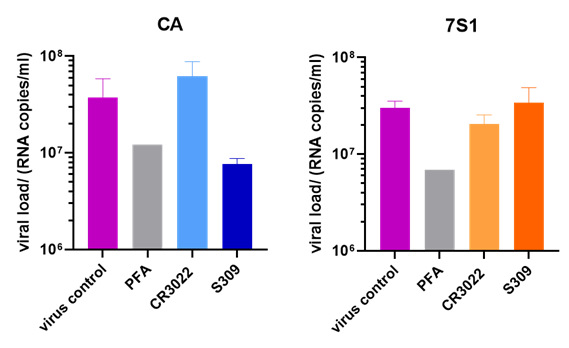


**Supplementary Figure 4. Susceptibility of SARS-CoV-2 strains to inhibition by monoclonal antibodies in 293T cells.** Replication of SARS-CoV-2 input virus (CA, left panel) and S309-resistant strain (7S1, right panel) in HEK293T cells in the presence of CR3022 and S309 at a concentration of 5 µg/ml. Controls included HEK293T cells infected in the absence of monoclonal antibodies (virus control) and paraformaldehyde (PFA)-fixed HEK293T cells to quantify background viral load. Data show mean and standard deviation of one experiment performed in triplicates.

# References

1. Baker NA, Sept D, Joseph S, Holst MJ, McCammon JA. Electrostatics of nanosystems: application to microtubules and the ribosome. Proc Natl Acad Sci U S A. 2001;98(18):10037-41.
